# Bio-Based Polymers for Engineered Green Materials

**DOI:** 10.3390/polym12040775

**Published:** 2020-04-01

**Authors:** Gianluca Tondi, Thomas Schnabel

**Affiliations:** 1 Department of Land, Environment, Agriculture and Forestry, University of Padova, Viale dell’Università 16, 35020 Legnaro, Italy; 2 Forest Products Technology & Timber Constructions Department, Salzburg University of Applied Sciences, Marktstrasse 136a, 5431 Kuchl, Austria; thomas.schnabel@fh-salzburg.ac.at

Every. Single. Carbon atom oxides to CO_2_ at the end. Every. Single. One.

The more we pump petroleum-fixed carbon into the carbon cycle, the higher will be the concentration of CO_2_ in the atmosphere. The higher the CO_2_, the greater will be the temperature of the planet. This increase in temperature will also raise the concentration of water in air. Water and CO_2_ are the molecules that contribute the most to the greenhouse effect. The planet is subject to daily warming, and the later we take account of it and act, the more difficult will the battle against global warming be.

It is time to change our views and make this problem a priority. Consumer demands need to care about the carbon neutrality of products so that the market and industry are forced to offer alternative and more sustainable solutions. 

In polymer science, this message means that we are to exploit as much as possible the materials that nature synthesizes, process them in a sustainable way, and eventually modify them to give the new materials the high-performing properties we are used to. We aim to zealously maintain the carbon atoms that are fixed in solid phase.

This is what our scientific community is trying to do on a daily basis—taking important steps to produce carbon-neutral, bio-based, high-performing materials.

## Content of This Issue

**Figure 1:**
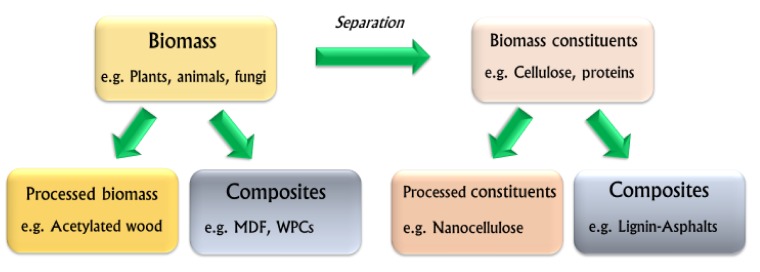
Diagram showing research topics in the field of bio-based polymers.

The current research on bio-based polymers is summarized in Figure 1. 

Among the different kind of biomasses, plants derivatives are the most widely investigated because they are by far the more abundant and are easy to source.

In this special issue, wood is treated to enhance its durability with alkali lignin at high temperature [1] and improve its transparency for the preparation of bleached thin translucent veneer [2]. This sustainable harvested biomass is also considered a source of molecules: a broad number of extractives such as taxifolin and larixol [3] were extracted by simple extraction, but organic oils could also be gained by applying thermal pyrolysis [4] for further application. 

With regard to biomass constituents, cellulose is certainly the more investigated subject in bio-polymer science. Despite pulping and paper-making having been used for centuries, there is a strong interest in improving the process and applying it to other biomasses, in an effort to enhance yield and pulp quality [5,6].

In recent years, scientific interest has moved to nano-fibrillated cellulose. In this issue, many research groups have proposed interesting technologic solutions [7]. Jiang et al. proposed an efficient method for obtaining NFC from Artemisia Vulgaris bast [8]. Kang et al. found a one-pot method to oxide it and use the product in cosmetic and biomedical applications [9], while other researchers have found attractive ways to modify the cellulose membrane by adding graphene oxide or grafting it to enhance its adsorption properties [10,11].

Cellulosic fibres were combined with various materials to enhance mechanical properties, not only in thermoplastics as polyamides, but also other bio-polymers such as polylactic acid and pectines [12,13,14,15,16,17].

Cellulose was also used as support for metals and other polymers such as polycaprolactone through electrospinning deposition [18,19].

Other research groups have exploited polysaccharides such as hemicelluloses, starch, and alginate to produce hydrogels, microparticles, and sponges for methylene blue removal [20,21,22]. Furthermore, bioplastics have been characterized, highlighting that starch has a higher recycle sensibility, while PLA is subject to a decrease in its molecular mass during reprocessing [23].

In spite of their extreme variability, proteins are a consistent subject of study in material science. In this volume, the growing mechanism of silkworm cocoons produced in confined spaces and the use of the silver coated peptone for anti-bacterial effects are presented [24,25].

We present three studies on polyhydroxyalkanoate produced by bacterial fermentation. These macromolecules are biodegradable polyesters and their synthesis is affected by several parameters, of which concentration of substrate and cycle length are fundamental [26]. These promising bio-material often have an unpleasant smell and limited mechanical properties, which can be reduced by adding organoclays [27,28].

Several interesting bio-based polymers from other resources and processes are also presented. Polyesters and NIPU were synthesized using vegetal oils, offering products with enhanced mechanical properties and high thermal stability, respectively [29,30]. Latex was exposed to photodegradation catalyzed by TiO_2_ to obtain easy-to-manage low molecular weight rubber [31]. In a study by Yu et al., the possibility to include heavy bio-oils in asphalt rubber to improve the rutting and fatigue resistance of the paving material is shown [32].

The polymerization of natural monomers such as tannins and dopamine were also exploited to produce high-absorbing materials and more mechanically stable explosives [33,34].

In summary, sustainably produced bio-based polymers can be studied from many aspects: We can consider the bio-materials, their constituents obtained by various processes, modifications, and their combinations in composites. Every time a researcher finds new ways to replace fossil-based resources to produce materials, the chances of keeping carbon atoms fixed to the material increases. We will, thus, soon be able to offer high-performing bio-based engineered products and trigger a green revolution.
